# Contrasting roles for actin in the cellular uptake of cell penetrating peptide conjugates

**DOI:** 10.1038/s41598-018-25600-8

**Published:** 2018-05-09

**Authors:** L. He, E. J. Sayers, P. Watson, A. T. Jones

**Affiliations:** 1 Cardiff School of Pharmacy and Pharmaceutical Sciences, Redwood Building, Cardiff University, Cardiff, Wales CF10 3NB UK; 2 Cardiff School of Biosciences, The Sir Martin Evans Building, Cardiff University, Cardiff, Wales CF10 3AX UK

## Abstract

The increased need for macromolecular therapeutics, such as peptides, proteins and nucleotides, to reach intracellular targets necessitates more effective delivery vectors and a higher level of understanding of their mechanism of action. Cell penetrating peptides (CPPs) can transport a range of macromolecules into cells, either through direct plasma membrane translocation or endocytosis. All known endocytic pathways involve cell-cortex remodelling, a process shown to be regulated by reorganisation of the actin cytoskeleton. Here using flow cytometry, confocal microscopy and a variety of actin inhibitors we identify how actin disorganisation in different cell types differentially influences the cellular entry of three probes: the CPP octaarginine – Alexa488 conjugate (R8-Alexa488), octaarginine conjugated Enhanced Green Fluorescent Protein (EGFP-R8), and the fluid phase probe dextran. Disrupting actin organisation in A431 skin epithelial cells dramatically increases the uptake of EGFP-R8 and dextran, and contrasts strongly to inhibitory effects observed with transferrin and R8 attached to the fluorophore Alexa488. This demonstrates that uptake of the same CPP can occur via different endocytic processes depending on the conjugated fluorescent entity. Overall this study highlights how cargo influences cell uptake of this peptide and that the actin cytoskeleton may act as a gateway or barrier to endocytosis of drug delivery vectors.

## Introduction

Cell penetrating peptides (CPPs) are a group of short sequences typically containing 5–30 amino acids that have been extensively investigated as carriers for intracellular delivery of various cargos including genetic material, peptides, proteins and nanoparticles^1–4^ Numerous efforts have been made to unveil the mechanisms of CPP translocation to the cytoplasm and cytosol of cells, and it is now well accepted that two modes of cell entry exist: direct membrane translocation, which may be energy and temperature independent, and uptake via one or more energy dependent endocytic pathways^5,6^. The propensity for uptake via these mechanisms is dependent on the peptide sequence, choice of cargo, *in vitro* model and can be influenced by experimental factors, including incubation temperature and the presence or absence of serum in media^7^. In a number of CPP studies an intact actin cytoskeleton has been proposed to be required for cell internalisation and CPPs inside and outside of cells can modify the actin cytoskeleton to influence cellular processes including CPP entry^8–11^.

One endocytic pathway that is absolutely reliant on actin is macropinocytosis. When activated this process has the capacity to form large plasma membrane derived intracellular vesicles termed macropinosomes^12–15^. Classically macropincytosis is induced in response to growth factor activation such as epidermal growth factor (EGF) binding to the EGF receptor, initially leading to extensive actin-dependent ruffling on the plasma membrane. This induces a “gulping effect” manifest as an increased uptake of extracellular fluid^13,14,16^. Much of the information known regarding growth factor induced and actin dependent macropinocytosis comes from studies on high EGFR expressing A431 skin epithelia cells and their response to EGF^13,17,18^. Of interest are observations that some CPPs under defined experimental conditions may induce plasma membrane effects similar to that seen upon growth factor activation^19–21^ and in line with this that they promote the concomitant uptake of dextran, a well characterised marker of fluid phase endocytosis^22–24^. Dextran itself, in addition to being widely used as a fluid phase endocytic probe has been extensively investigated as a drug delivery vector^25^.

Tools used routinely to examine the roles of the actin cytoskeleton in various cellular processes, including endocytosis and CPP entry are pharmacological/chemical inhibitors. The most notable such agent is the fungal metabolite cytochalasin D (Cyt D) which disrupts actin polymerisation and is a well characterised inhibitor of various endocytic mechanisms^26–28^. Other natural compounds and synthetic products such as Latrunculin B (Lat B) and Jasplakinolide (JAS) have been identified or developed to target the actin directly or indirectly and to disrupt its organisation and function^29^. Very few studies have investigated the effects of these other actin disrupters on CPP uptake though it is generally recognised that actin disruption universally inhibits CPP entry.

Here we show that the effects of actin disruption on uptake of CPPs and dextran is cell type dependant and in A431 skin epithelia, in complete contrast to HeLa cells, leads to a dramatic increase in uptake of EGFP-R8 and dextran but inhibits the uptake of R8-Alexa488. Together the data indicate that actin organisation has very different influences on uptake of these octaarginine and fluid phase conjugates and that actin could be targeted to enhance cellular uptake of drug delivery vectors.

## Materials and Methods

### Cell culture

HeLa (cervical carcinoma, epithelial) cells (ATCC Code CCL-2) or A431 (skin epidermal) cells (ATCC Code CRL-1555) were maintained in growth Dulbecco’s Modified Eagle’s medium (D-MEM) supplemented with foetal bovine serum (10%, final volume) and penicillin/streptomycin (100 units/ml and 100 μg/ml respectively), herein referred to as complete medium in a humidified 5% CO_2_/37 °C incubator as a subconfluent monolayer. Cells were obtained from the ATCC and were routinely tested for mycoplasma.

### Peptide Labelling

Peptide octaarginine (R8), extended with GC at its C-terminal (RRRRRRRR-GC), was synthesized by American Peptide Company (California, USA) and labelled with Alexa Fluor^®^ 488 C5 maleimide salt (Invitrogen, Paisley, UK) as previously described^30^. Labelling and conjugate mass (2126.5 Da) was confirmed by matrix-assisted laser-desorption ionization–time-of-flight spectrometry and quantified by UV spectrometry. All labelled peptides were resuspended to a stock concentration of 1 mM in autoclaved distilled water, aliquoted and frozen at −80 °C until needed.

### Expression and purification of recombinant proteins

Plasmids encoding histidine tagged - EGFP-octaarginine, or - EGFP referred to here as EGFP-R8 or EGFP, were a kind gift from Professor Shiroh Futaki, University of Kyoto, Japan. To produce the plasmid the DNA fragment encoding EGFP was amplified from pEGFP-N1 (Clontech) using primers possessing the sequences for NdeI followed by His_6_, and R8 and stop codon followed by EcoRI. The fragment was cleaved by NdeI and EcoRI and cloned into a pET3b (Novagen) derivative without N-terminal T7.Tag sequence to construct *E. Coli* expression vector pEV3b^31^ that has NdeI and EcoRI sites within the multi cloning site. Proteins were produced in *E. coli* BL21 (DE3) cells and assessed for purity and molecular weight as described in Supplementary Information and shown in Supplementary Fig. 1.

### Live cell imaging of endocytic probes and R8 conjugates

HeLa or A431 cells were seeded on 35 mm MatTek dishes (Ashland, US) at respective densities of 5 × 10^5^ cells/dish and 1.0 × 10^6^ cells/dish and maintained in 2.5 mL D-MEM containing 10%, 100 IU/mL penicillin and 100 µg/mL streptomycin. Cells were then allowed to adhere for 24 hr at 37 °C/5% CO_2_ to reach a confluence of ~80%. On the day of experimentation, they were washed with PBS and then incubated with 100 µl serum-free D-MEM (thus reducing well described serum binding effects^32^) containing either 2 µM CPP conjugate (R8-Alexa488 or EGFP-R8), 5 µg/ml transferrin-Alexa 647 (TF-647) or 0.1 mg/ml dextran (10 kDa)-Alexa 647 (Dex-647). The cells were then incubated for 1 hr (or 30 min for TF-647) at 37 °C/5% CO_2_ before washing 3x with PBS (TF-647 or Dex-647) or PBS 0.5 mg/mL heparin (R8-conjugates). They were then washed once with PBS, prior to the addition of 2 mL of imaging medium (phenol red free RPMI, 20 mM HEPES pH 7.4) and analysis by live cell confocal microscopy.

### Actin inhibition and confocal microscopy of endocytic probes and R8 conjugates

Cells were seeded on MatTek dishes as previously described and were washed with PBS before preincubation at 37 °C with diluent control (DMSO) or actin inhibitors: 10 µM Cyt D for 15 min; 0.1–1 µM Lat B for 30 min; 0.2–4 µM JAS for 45 min; 50–100 µM Y27632 for 4 hr in serum-free D-MEM at 37 °C/5% CO_2_. Endocytic probes (TF-647, Dex-647) or R8-conjugates were then incubated with the cells in the absence (control) or presence of the inhibitors and further incubated at 37 °C/5% CO_2_ for times specified in figure legends prior to analysis by confocal microscopy. For pulse chase experiments, A431 cells were incubated with 0.1 mg/ml Dex-647 for 2 hr (pulse) followed by washing and a further 4 hr (chase) in fresh complete growth medium. EGFP-R8 (2 µM) was then added for 1 hr in the absence or presence of 10 µM Cyt D. EGFP-R8 and Dex-647 subcellular distribution was then analysed in live cells using confocal microscopy. For low temperature experiments, cells were preincubated with Cyt D as above and then placed on ice for 2 min prior to addition of ice cold EGFP-R8. The cells were then maintained under these conditions for 1 hr prior to analysis by confocal microscopy.

### Confocal microscopy

Analysis was performed on a Leica SP5 confocal laser scanning microscope, equipped with laser lines: 405 Blue Diode (Excitation wavelength 405 nm for Hoechst 33342) Argon (Excitation wavelength 488 nm for Alexa 488 or EGFP), Helium Neon Laser 1 (Excitation wavelength 543 nm for Tetramethyl-Rhodamine Phalloidin (Rh-P)) and Laser 2 (Excitation wavelength 633 nm for Alexa 647 conjugates). All images presented in this study were obtained using a HCX PL APO 63x 1.4 NA oil immersion objective with Leica Type F immersion oil. Parameters “Gain” and “Off-set” of the individual photomultiplier tubes were adjusted between experiments in order to obtain optimal image acquisition avoiding saturation. For multi-channel image acquisition, the channels were scanned in a sequential recording mode to avoid spectral cross-talk caused by overlapping excitation and/or emission spectra of fluorophores. Acquisition mode “XYZ” and image resolutions (pixels/image) of 512 × 512 or 1024 × 1024 were selected and pinhole size was set to 1 Airy Unit. Depending on acquisition requirements the scan speed was set as either 400 or 700 Hz and for single section images, the same optical section was scanned 3x to generate a line average image. Captured images were then analysed using ImageJ. In some cases (live cell imaging) the same cells were also imaged using Differential Imaging Contrast microscopy (DIC).

### Actin cytoskeleton visualisation

A recently described method was utilised giving information on actin architecture at different regions of the cells termed basal and cell body and apex (CBA). For actin staining, HeLa (0.5 × 10^6^ cells/well) or A431 (1.0 × 10^6^ cells/well) cells in 2.5 mL D-MEM containing 10% FBS were seeded on round borosilicate No.1.5 cover slips in 6 –well plates and allowed to adhere for 24 hr at 37 °C/5% CO_2_, to reach a confluence of ~80%. The cells were then treated with actin disrupters as described above, washed 3x with PBS and fixed for 15 min at room temperature with 3% PFA in PBS. Cells were then washed 3x with PBS and permeabilised with 0.2% Triton X-100 in PBS at room temperature for 5 min, washed 3x with PBS and stained for cell nuclei with 1.0 µg/ml Hoechst 33342 in PBS and for polymerised actin with 1.0 µg/ml Rh-P in PBS for 15 min at room temperature. The cells were finally washed 3x with PBS and once with distilled water, coverslips were mounted in DAKO mounting medium on glass microscope slides and imaged by confocal microscopy^19^.

### Flow Cytometry

A431 cells were seeded onto 12-well plates and cultured for 24 hr to be ~80% confluent on the day of experiment. Cells were washed twice with PBS and then incubated with diluent control (DMSO/dH_2_O) or actin inhibitors as previously described. They were then loaded with serum-free DMEM containing either 2 µM EGFP-R8, 5 µg/ml TF-647 or 0.1 mg/ml Dex-647 in the presence or absence of actin inhibitors for times stipulated in the figure legends. The plates were then placed on ice to prevent further uptake and washed 3x with either ice-cold PBS or 0.5 mg/ml heparin in PBS. To remove the plasma membrane bound TF, cells were washed 3x with ice-cold PBS followed by an incubation for 1 min in ice-cold acid wash (0.2 M acetic acid, 0.2 M NaCl, pH 2.0)^33^. All cells were then washed twice with PBS at room temperature before trypsinisation at 37 °C for 5 min before placing the detached cells in centrifuge tubes and centrifuging at 4 °C for 3 min at 800 × *g*. The obtained cell suspension was washed 3x with ice-cold PBS and finally resuspended in 300 µl ice-cold PBS for measurement of cell-associated fluorescence (cell gate 50,000 cells) by flow cytometry on a BD FACSVerse utilising a 488 laser and 527/32 filter for EGFP analysis and a 633 nm laser and 660/10 filter for Alexa 647. Cells were gated using FSC-A/SSC-A to isolate single cell populations and analysed using FlowJo.

### Statistics

Flow cytometry measurements represent the mean fluorescence from three independent experiments performed in duplicate, where each individual data point was the geometric mean. A two-tailed unpaired Students t-test was used to test for differences within experiments. Significant differences were classed as having a P value of < 0.05.

### Data Availability

The datasets generated during the current study are available from the corresponding author on reasonable request.

## Results

### Cyt D effects on actin and endocytosis in HeLa and A431 cells

Cyt D binds to the fast-growing ends of actin nuclei and filaments, preventing addition of monomeric actin to these sites. In our previous studies using flow cytometry we showed that Cyt D inhibited the uptake of Alexa488-CPPs R8 and Tat in two different cell models, HeLa and A431^33^. But interestingly, the effect of actin disruption with this agent on the uptake of dextran was highly dependent on the choice of cell line as there was no effect in HeLa cells and an increased uptake in A431 cells. Here we further explored these contradictory effects by also performing uptake studies with recombinant EGFP-R8 following purification from *E. Coli* as described in Supplementary methods and Supplementary Fig. 1.

**Figure 1:**
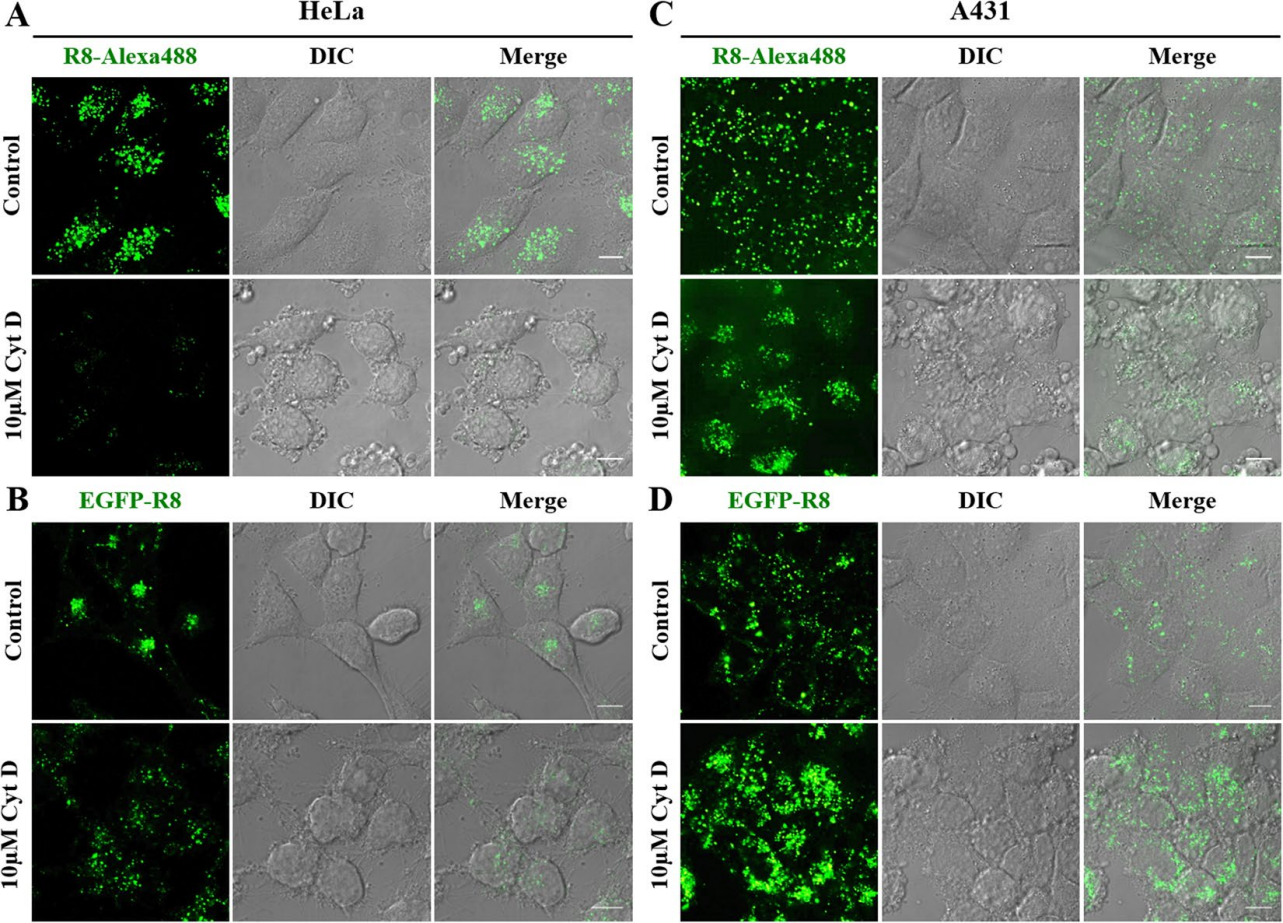
Cytochalasin D effects on the cellular uptake of R8 conjugates in HeLa or A431 cells. HeLa (**A,B**) or A431 (**C,D**) were preincubated with 10 µM Cyt D or diluent control for 15 min prior to incubation with 2 µM R8 conjugates (R8-Alexa 488, **A**,**C**, or EGFP-R8, **B,D**) for 1 hr in the presence or absence of 10 µM Cyt D before washing in heparin and analysis by confocal microscopy. Shown are single projection images of fluorescence only (R8-Alexa 488/EGFP-R8), DIC and merges of fluorescence and DIC of the same cells. Scale bars 10 µm.

**Figure 2:**
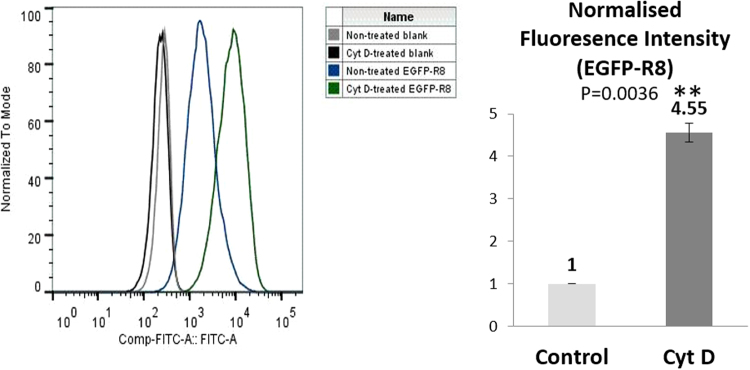
Cytochalasin D treatment increases the cellular uptake of EGFP-R8 in A431 cells. Cells were preincubated with 10 µM Cyt D or diluent control for 15 min prior to incubation with 2 µM EGFP-R8 in the absence or presence of 10 µM Cyt D for 1 hr. Cells were then washed, trypsinised and analysed by flow cytometry. Data represent the geometric means ± S.D. from three independent experiments.

At 2 µM extracellular concentration, both R8 conjugates, in the two cell lines, labelled vesicular structures scattered throughout the cytoplasm as expected (Supplementary Figs 2 and 3), no cell associated fluorescence was observed for EGFP alone. We then performed the same experiments in cells incubated with Cyt D, focusing on effects on endocytosis and also cell morphology. 10 μM of the inhibitor was used as this concentration has previously been shown by different groups to inhibit the uptake of cationic CPPs such as HIV-Tat and R8 linked to different cargoes^33–35^. DIC microscopy highlighted the dramatic morphological effects on both cell lines incubated with this agent (Fig. 1 and Supplementary Fig. 4). Cyt D inhibited the internalisation of R8-Alexa 488 in HeLa cells (Fig. 1A), confirming our published data using flow cytometry^33^. There was no clear evidence of Cyt D mediated inhibition of EGFP-R8 but the drug caused a scattering of the fluorescent structures compared to untreated cells that usually displayed strong juxtanuclear labelling (Fig. 1B). In contrast to HeLa cells there was less of a noticeable reduction in uptake of R8-Alexa488 in A431 cells but a clear change in subcellular distribution from evenly scattered throughout the cytoplasm in control cells to a largely clustered phenotype in treated cells (Fig. 1C). In contrast to the results obtained with R8-Alexa 488, Cyt D dramatically enhanced the internalisation of EGFP-R8 into small and large cytoplasmic structures (Fig. 1D). This data was confirmed in A431 cells using flow cytometry showing a highly significant 4.5 fold increase in intracellular fluorescence (Fig. 2), P = 0.0036.

In these two cell lines, we also investigated the effects of Cyt D on the uptake of dextran, that enters cells via fluid phase uptake, and transferrin that enters via clathrin mediated endocytosis. In agreement with our previous flow cytometry and microscopy studies with 40 KDa FITC and Alexa488 dextran^33^, uptake of dextran-Alexa647 was dramatically increased in A431 cells (Supplementary Fig. 5A, C).

Cell associated transferrin fluorescence was not markedly different between control and Cyt D treated HeLa cells (Supplementary Fig. 5B) confirming previous reports performed at the same concentration in this cell line^36^. Transferrin fluorescence in Cyt D treated HeLa cells was, however, more prominent on the plasma membrane compared to a strong perinuclear enrichment of fluorescence in control cells. Flow cytometry utilising acid washing of surface label confirmed that there was no difference in fluorescence uptake of transferrin in A431 cells treated with this drug (Supplementary Fig. 6), confirming previous studies in this cell line^27^. We focused our further investigations on the A431 cell line and EGFP-R8, as this was the conjugate that gave unexpected Cyt D mediated uptake effects.

To identify whether the enhanced cellular uptake of EGFP-R8 in A431 cells by Cyt D treatment was an energy dependant process, the same experiments were performed with cells pre-incubated at 37 °C with the drug, then placed on ice and maintained at 4 °C throughout the subsequent incubation with EGFP-R8. There was very little evidence above background fluorescence that EGFP-R8 entered cells at 4 °C in the presence or absence of Cyt D (Supplementary Fig. 7) confirming that the enhanced EGFP-R8 internalisation induced by the inhibitor was energy dependent.

To try and identify the subcellular compartments occupied by EGFP-R8 we performed pulse/chase experiments with Dex-647: a 2 hr pulse labelled endolysosomal organelles was followed by a 4 hr chase to specifically label late endosomes and lysosomes^37^. EGFP-R8 was then incubated with the cells for 1 hr in the presence or absence of Cyt D. Control cells (Supplementary Fig. 8A, top row, indicated by arrows) show colocalisation of the two fluorophores demonstrating trafficking of some EGFP-R8 to dextran labelled late endosomes and lysosomes. However, EGFP only structures were also clearly visible suggesting that this fraction had not yet entered these structures or were trafficking to a different location. To verify that the EGFP fluorescence was being emitted from inside the cells we captured images of the Cyt D treated cells through the Z axis starting 1.44 µm from the glass surface to a height of 14.5 µm. This allowed us to visualise the dextran and EGFP-R8 structures at different focal planes and the data confirmed that the EGFP fluorescence was of intracellular origin (Supplementary Fig. 8B).

### Effects of other actin reagents on actin and cellular uptake of EGFP-R8

**Figure 3:**
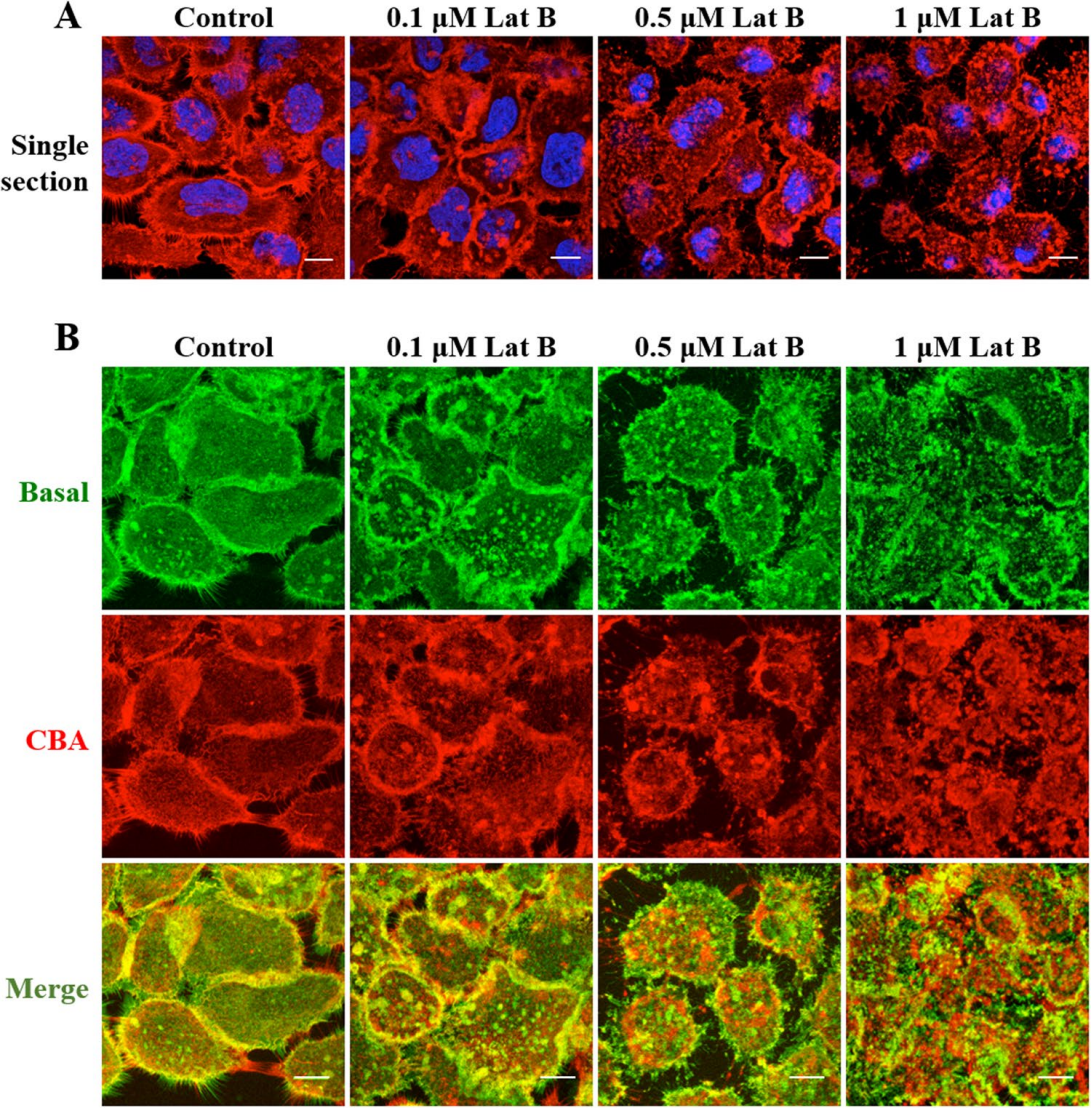
Effects of Lat B on the actin architecture in A431 cells. Cells on coverslips were treated either with diluent control (0.1% DMSO) or 0.1–1.0 μM Lat B for 30 min before fixing and staining with Rh-P and Hoechst. Single sections (**A**) show the overall distribution of the actin relative to the nucleus. (**B**) Actin arrangement from the basal, CBA regions and composite (Merge) of basal and CBA. Scale bars 10 μm.

Lat B, binds to and complexes with actin monomers at a 1:1 molar ratio and prevents polymerisation^38^. We initially investigated the concentration dependent effects of this drug on the actin cytoskeleton. At the lowest concentration (0.1 μM) the drug caused the formation of small actin aggregates at both the basal and cell body and apex (CBA) sections of the cells (Fig. 3). At 0.5 μM, these aggregates were extremely prominent especially at the basal section. At 1 μM the drug caused major disruption of the actin organisation manifest as amorphous structures scattered throughout the cytoplasm. The effects of this drug on cell morphology is further highlighted in Supplementary Fig. 4.

**Figure 4:**
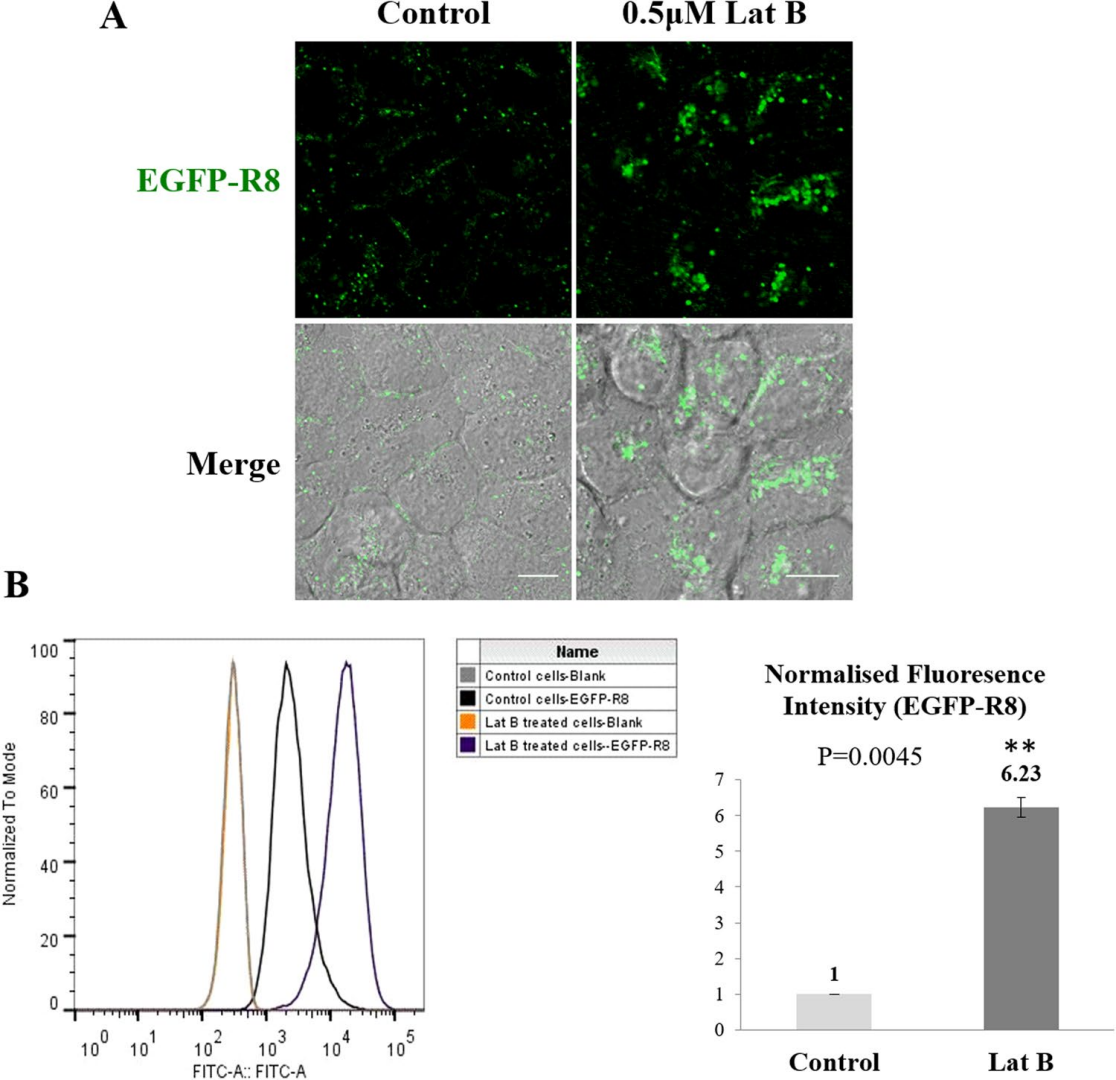
Effects of Lat B on the cellular uptake of EGFP-R8 in A431 cells. Cells were preincubated with either with diluent control or 0.5 μM Lat B for 30 min prior to incubation with 2 µM EGFP-R8 for 1 hr in the absence (control) or presence of 0.5 µM Lat B and washed in heparin. (**A**) Cell associated fluorescence was analysed using confocal microscopy and shown are single projection images of fluorescence only (top rows) and merges of fluorescence and DIC of the same cells. Scale bars 10 µm. (**B**) Cells treated as in A were washed, trypsinised and analysed by flow cytometry. Data represent the mean of geometric means ± S.D. from three independent experiments.

We subsequently performed CPP uptake experiments in A431 cells pre-incubated with 0.5 µM Lat B prior to addition of EGFP-R8. The effect of Lat B (Fig. 4) was comparable to that of Cyt D (Fig. 2): the inhibitor treated cells showing much higher fluorescence compared to controls (Fig. 4). Flow cytometry analysis confirmed these findings and the 6.23 fold increase in uptake was highly statistically significant (P = 0.0045).

**Figure 5:**
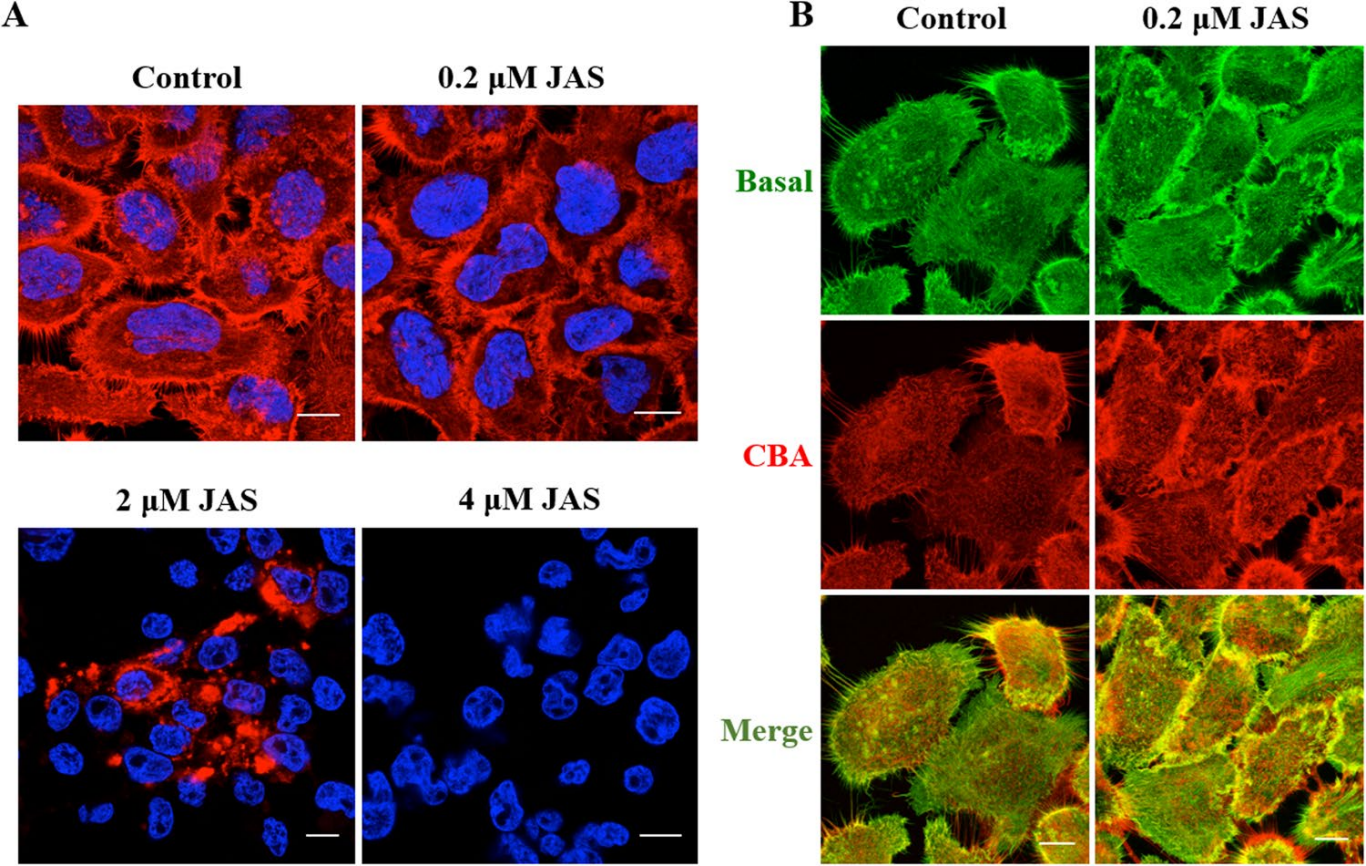
Effects of JAS on the actin architecture in A431 cells. Cells on coverslips were treated either with diluent control (0.1% DMSO) or 0.2, 2.0 or 4.0 μM JAS for 45 min before fixing and staining with Rh-P and Hoechst. (**A**) Single sections show the overall distribution of the actin relative to the nucleus. (**B**) Actin arrangement from the basal, CBA regions and composite (Merge) of basal and CBA. Scale bars 10 μm.

An alternative actin inhibitor, Jasplakinolide (JAS) binds directly to filamentous actin and unlike Cyt D and Lat B induces stabilisation of the existing actin filaments thus inhibiting actin disassembly^39–42^. Compared with control cells, no visible changes in actin organisation were observed in both basal and CBA sections of cells until the concentration of JAS reached 0.2 µM (data not shown and Fig. 5). At this concentration, actin staining was more prominent on the cell periphery at both basal and CBA regions. At 2 µM JAS the actin staining appeared in large clumps, previously identified as aggresomes^43^, or was absent, and was completely lost from all cells treated at 4 µM (Fig. 5A). This loss of fluorescence may be due to the fact that this drug has been shown to be a competitive inhibitor of phalloidin-actin binding^39^. Supplementary Fig. 4 shows the significant morphological effects of this drug on the cells.

**Figure 6:**
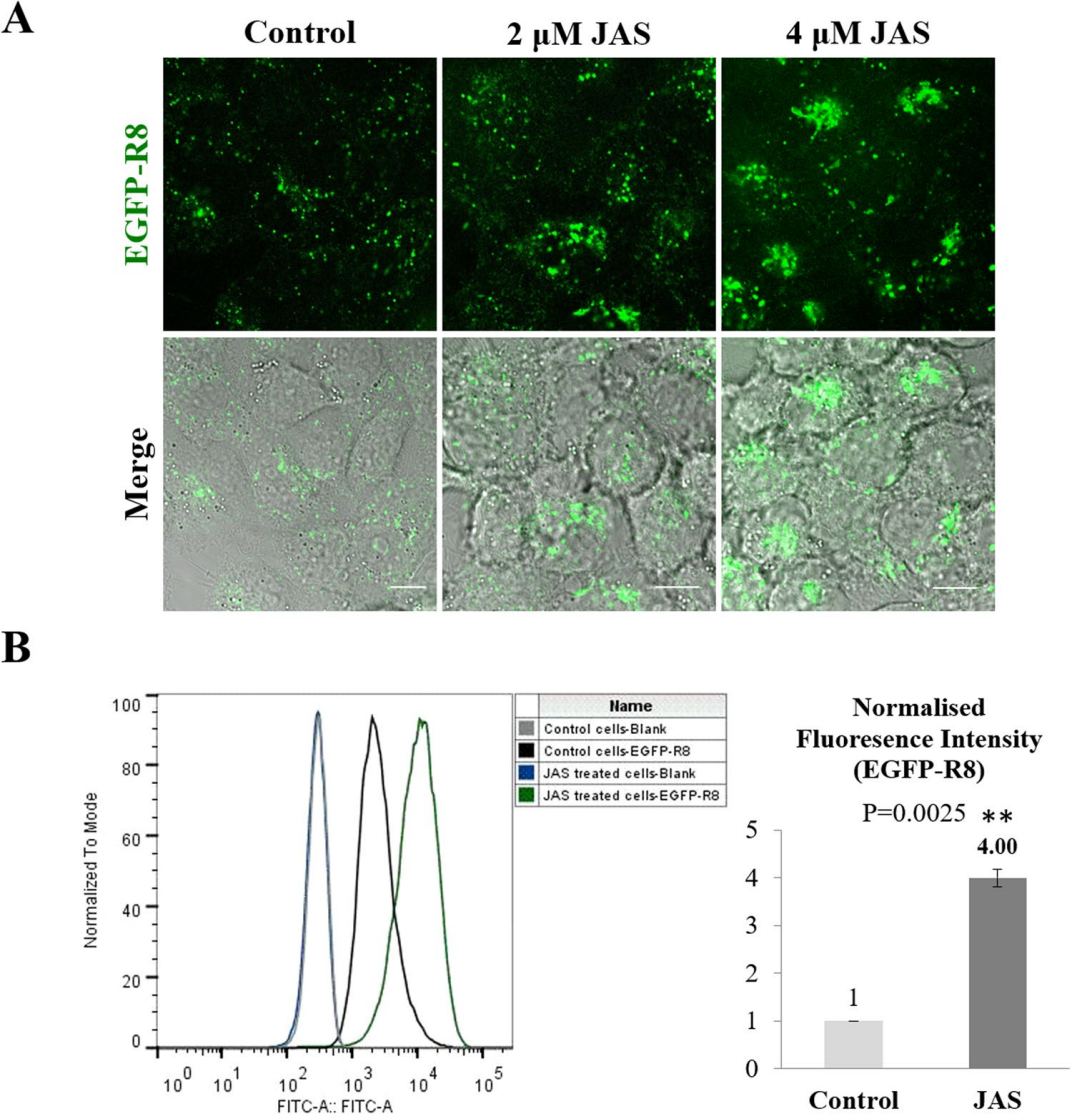
Effects of JAS on the cellular uptake of EGFP-R8 in A431 cells. Cells were preincubated with diluent control or 2–4 µM JAS for 45 min prior to incubation with 2 µM EGFP-R8 for 1 hr in the absence (control) or presence of 2–4 µM JAS and washed thoroughly using heparin. (**A**) Cell associated fluorescence was analysed using confocal microscopy and shown are single projection images of fluorescence only (top row) and merges of fluorescence and DIC of the same cells. Scale bars 10 µm. (**B**) Cells treated as in A with 4 µM JAS were then washed, collected and analysed by flow cytometry. Data represent the mean of geometric means ± S.D. from three independent experiments.

Confocal microscopy dramatic increase in EGFP-R8 uptake in JAS treated cells (Fig. 6A) and the drug caused a pronounced clustering of fluorescence in the cytoplasm. Equivalent experiments analysed by flow cytometry (Fig. 6B) indicated that JAS induced a four-fold increase in fluorescence that was statistically significant (P = 0.0025). The diluent control experiments with JAS required adding DMSO to the cells at a final concentration of 0.4% v/v; the highest used in this study. This concentration did not affect uptake of EGFP-R8 or R8-Alexa488 (Supplementary Fig. 9).

**Figure 7:**
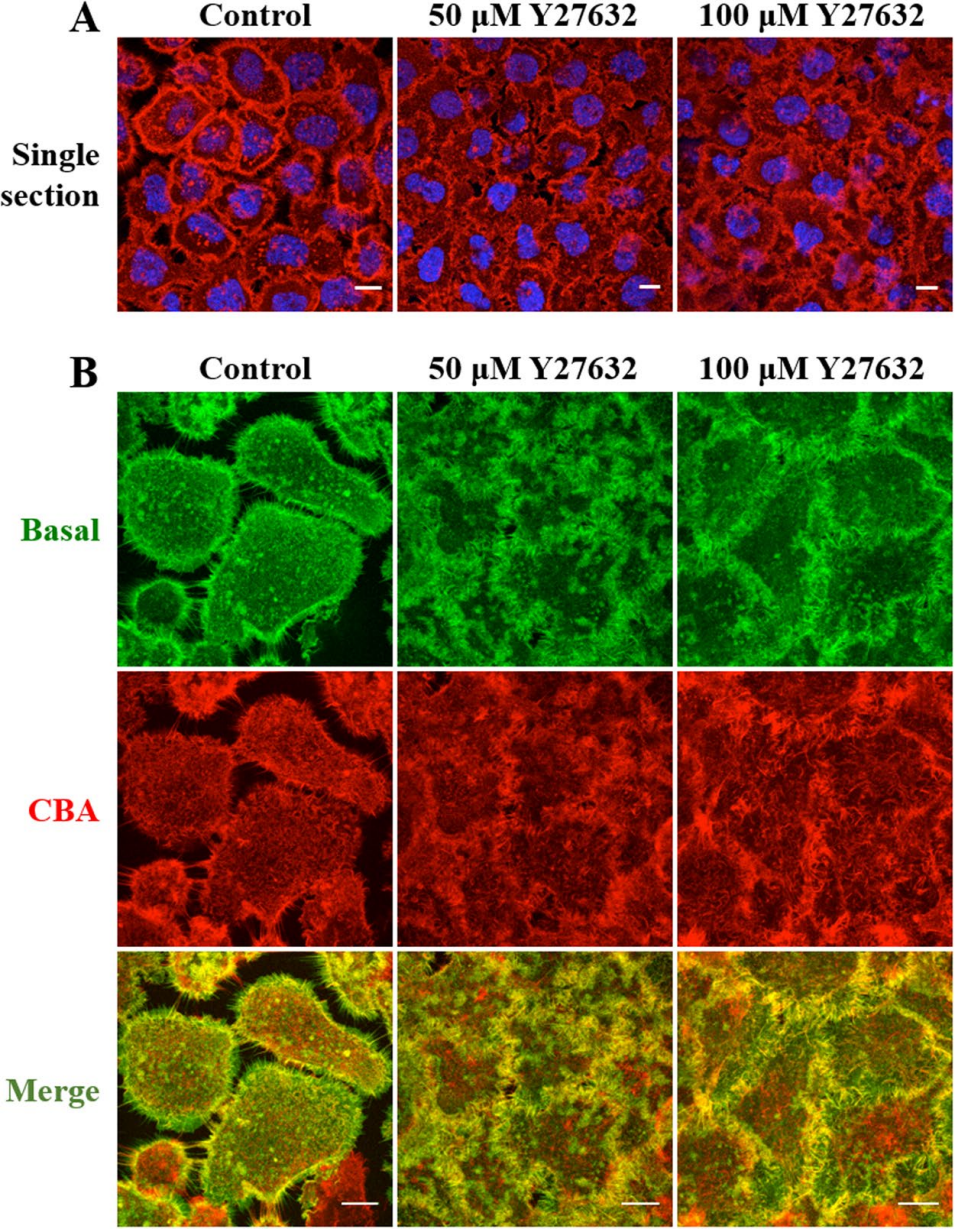
Effects of Y27632 on the actin architecture of A431 cells. Cells on coverslips were treated either with diluent control (D-MEM) or 50, 100 μM of Y27632 for 4 hr before fixing and staining with Rh-P and Hoechst. (**A**) Single sections show the overall distribution of the actin relative to the nucleus. (**B**) Actin arrangement from the basal, CBA regions and composite (Merge) of basal and CBA. Scale bars 10 μm.

Actin dynamics and regulation are achieved through the concerted action of actin regulatory proteins whose activities are finely modulated by protein kinases. These include Rho-associated coiled-coil forming serine/threonine kinases ROCK I and II that have been identified as important downstream effectors of RhoA through their interaction with its GTP-bound form^44,45^. A number of chemical inhibitors of the Rho-ROCK signalling cascade have been developed, including compound Y27632 [(+)-(R)-trans-4-(1-aminoethyl)-N-(4-pyridyl) cyclohexanecarboxamide dihydrochloride]. This has been shown to inhibit the kinase activity of a number of ROCKs by binding to their catalytic sites^46,47^. Initially, 1 and 10 μM concentrations of Y27632 were investigated on actin localisation based on published observations showing actin deformities at these concentrations^46,48^. Supplementary Fig. 10 demonstrates that no visible changes in the organisation of the actin cytoskeleton were detected up to 10 μM drug. Higher concentrations caused extensive actin deformities including the formation of a large number of actin needles, the majority of which ran perpendicular to the plasma membrane (Fig. 7).

**Figure 8:**
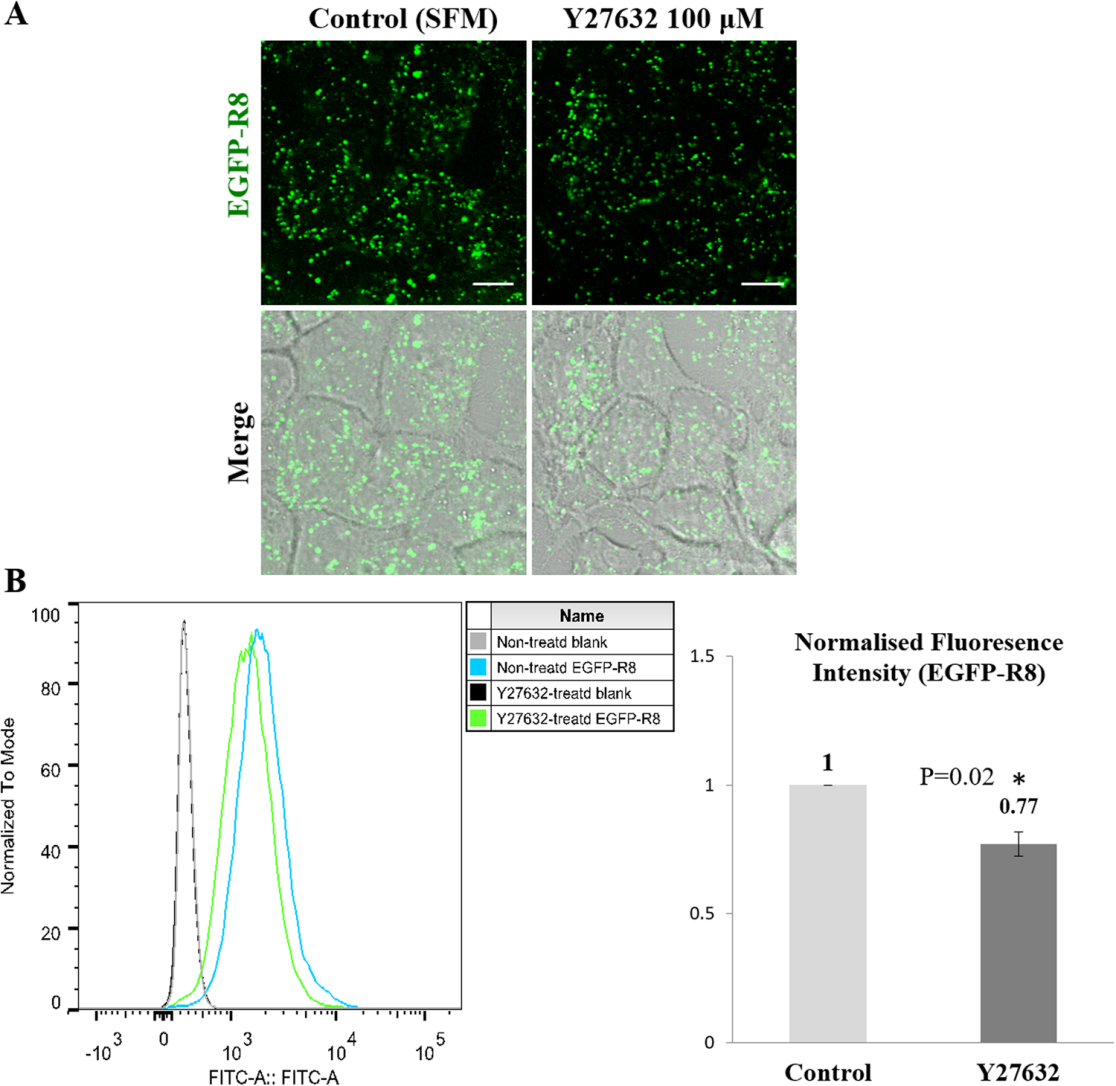
Effects of Y27632 on the cellular uptake of EGFP-R8 in A431 cells. Cells were preincubated with diluent control or 100 µM Y27632 for 4 hr prior to incubation with 2 µM EGFP-R8 for 1 hr in the absence (control) or presence of 100 µM Y27632 and washed with heparin. (**A**) Cell associated fluorescence was analysed using confocal microscopy and shown are single projection images of fluorescence only and merge of fluorescence and DIC of the same cells. Scale bars 10 µm. (**B**) Cells were washed, trypsinised and analysed by flow cytometry. Data represent the mean of geometric means ± S.D. from three independent experiments.

In CPP uptake experiments, Y27632 treated cells displayed scattered EGFP-R8 fluorescence in small vesicles in contrast to the vesicle aggregates seen in cells incubated with the other actin disrupting agents (Figs 1,4 and 8A) while DIC microscopy did not show the gross morphological effects caused by the other actin inhibitors. Flow cytometry analysis indicated that cell uptake of EGFP-R8 uptake was significantly inhibited by Y27632 but only by ~20% (Fig. 8B P = 0.02). To exclude the possibility that any of the actin inhibitors was affecting plasma membrane permeability, we incubated A431 cells with the inhibitors at the concentrations and incubations used above in the presence of live cell impermeable dye - DRAQ7. Using Tritin X-100 as positive control, none of the inhibitors showed any evidence of inducing plasma membrane permeabilisation (Supplementary Fig. 4).

## Discussion

Since the discovery of CPPs, considerable efforts have been made to elucidate their internalisation mechanisms. Focusing mainly on arginine rich variants, macropinocytosis has been proposed to be a major contributing pathway for delivery of low molecular weight fluorophores or larger cargo by these peptides^20,24,49^. This is based on studies utilising chemical macropinocytosis inhibitors including, and almost exclusively Cyt D for actin inhibition. It should however be noted that the involvement of actin activity has been shown for other endocytic pathways such as clathrin-mediated endocytosis (CME)^27,50^. As shown here and in several other studies, Cyt D at concentrations >5 µM, completely destroys actin organisation and, unsurprisingly, cell morphology is also compromised. A number of other natural compounds and synthetic products have been identified or developed to target the actin cytoskeleton directly or indirectly and to disrupt its arrangement/function. These include Lat B, JAS and Y27632 and these inhibitors have not been used to support Cyt D data for drug delivery endocytosis studies, including CPPs. To our knowledge this is the first study that has analysed CPP uptake with actin inhibitors employing different mechanisms of action.

By employing high content analysis of the actin architecture using confocal microscopy and then flow cytometry we were able to correlate actin disorganisation with cellular uptake of EGFP-R8 as a model protein-CPP conjugate. EGFP has been shown to be delivered into cells by R8 and R9^35,51^ but correlative information regarding the uptake mechanism of the proteins relative to that of the peptides attached to fluorophores is lacking. EGFP-Tat uptake has been proposed to be mediated via macropinocytosis and caveolae^24,52^. Both Tat peptide, octaarginine,  and shorter number of arginine residues (R7, R9) have also been shown to deliver proteins into cells to mediate biological effects^53–55^.

In this study, HeLa cells reacted in a somewhat predictable manner in response to actin breakdown with Cyt D showing a reduction in uptake of R8-Alexa488 and dextran. In this cell line EGFP-R8 uptake was also inhibited by Cyt D treatment suggesting a similar mechanism of uptake. We have previously shown that cortical actin in A431 cells is particularly prominent and suggested that this may have a role in the uptake of dextran that, under normal conditions, enters cells via constitutive pinocytosis; also termed fluid phase endocytosis. This can be differentiated from stimulus induced macropinocytosis^56,57^. This potential for the involvement of cortical actin was identified by discovering a rather unexpected and highly significant increase in the uptake of 10 and 40 KDa dextran in Cyt D treated A431 cells that was not observed with Alexa conjugates of transferrin, Tat and R8^33^.

In this study in we show that dextran and also EGFP-R8 uptake is significantly increased in A431 treated with Cyt D and contrasts to that observed for R8-Alexa488 (inhibition) and transferrin (no change). This strongly suggests that the process by which these two R8 conjugates interact with and/or enter cells is different. It should be noted that the structure of both conjugates differ in their cargo-CPP orientation and also the linkers utilised between them. We consider that it is highly unlikely that these factors mediate the differences in endocytic profiles of these conjugates. Due to the similarities we see in entry for dextran and EGFP-R8, we hypothesise that the differences are driven by the cargo and not the orientation of the CPP. But this warrants further study, noting that EGFP alone does not enter these cells at the concentrations used here, demonstrating a clear role for R8 that may be interacting differently with cells when attached to a fluorophore or protein. In none of the experiments performed did we observe clearly defined cytosolic EGFP fluorescence in Cyt D treated cells demonstrating that the effects of this drug maintained the localisation of the protein within a membrane bound compartment.

We have previously shown that R8 and Tat peptides as Alexa conjugates traffic rapidly by endocytosis to reach lysosomes^30^ and by performing pulse chase experiments we now show that the same is true for EGFP-R8, demonstrating that it is trafficked to the same degradative compartments. From these combined studies we also highlight that traffic to lysosomes of R8 attached to different cargo is also entirely dependent on functional actin.

Our findings prompted us to further study EGFP-R8 uptake in cells treated with inhibitors of actin that operate via different mechanisms and affect actin dynamics in different ways. Like Cyt D, at effective actin disrupting concentrations, JAS and Lat B also affected cell morphology and increased EGFP-R8 uptake that was again highly enriched in large structures in particular regions of the cells. Previous studies in non-polarised epithelial MDCK cells demonstrated that JAS treated cells had much higher levels of internalised and retained dextran compared with control cells^57^. In polarised cells, however, this effect was limited to dextran entering via the basolateral surface and the retained dextran was observed in large structures as we observe for EGFP-R8. It remains to be determined whether EGFP-R8 would also behave in a similar manner when introduced to different surfaces of polarised cells and whether this is influenced by any differences in cortical tension between these surfaces. The nature of these large structures is unknown but may be formed by tubulation of the plasma membrane that has been shown to be caused by Cyt D^58^. The plasma membrane tension of mammalian cells is coordinated and maintained through the actin network, and this profoundly influences endocytosis^59,60^. The reduced cortical tension resulting from actin disruption in MDCK basolateral surfaces^57^ and here in A431, cells may favour the formation of invaginations and tubulation to allow entry of macromolecules. This strongly supports studies showing an increased uptake of 70 KDa dextran and the folic acid receptor in Cyt D treated cells^61,62^. Both folic acid and dextran have been extensively utilised as, respectively, targeting and passive vectors for drug delivery^25,63^.

Through interaction with the Rho GTPase, ROCKs play a central role in actin network assembly and membrane association^64^. In A431 cells the effects of Y27632 on the actin cytoskeleton was more subtle than that observed with the agents that directly targeted actin. No change in cell morphology was observed in treated cells but actin distribution was very different at high 100 µM concentrations. Effects of this agent in human trabecular meshwork eye cell actin has been shown to be extremely prominent at much lower concentrations, showing a loss of filamentous actin^65^. In contrast to results observed with direct actin inhibitors, we observed a slight but significant decrease in the uptake of EGFP-R8 in cells treated with Y27632. To our knowledge no CPP studies have been performed with this drug though it has previously been shown to affect the internalisation of plasma membrane receptors and also to inhibit (~50%) the uptake of 70 KDa dextran in macrophages^66–68^. This again supports our suggestion that dextran and EGFP-R8 uptake is responsive to the same inhibition in A431 cells but that they may not necessarily enter via the same mechanism. This supports recent observations showing that cell uptake profiles of HIV-TAT peptide and dextran can be separated^69^.

## Conclusions

Interpretation of endocytosis data in *in vitro* models obtained using actin modifying agents is difficult as the network that this protein nucleates is a regulator of so many processes; global cell morphology is often affected by its perturbation. Here we highlight that disruption of actin in some cell types promotes cell uptake of specific molecules whilst having no effects, or even inhibitory effects on others. From careful scrutiny of the literature there is precedent for this in other cell types and distinct plasma membrane regions in polarised cells. The possibility exists that these cells have a specific but common cortical organisation of actin that could be exploited to drive internalisation of drug delivery vectors carrying macromolecular therapeutics.

## Electronic supplementary material

Supplementary Information
