# Supplementary material for: Contrasting roles for actin in the cellular uptake of cell penetrating peptide conjugates
Source: Sci Rep. 2018 May 9;8:7318. doi: 10.1038/s41598-018-25600-8 (PMC5943252; doi:10.1038/s41598-018-25600-8)
Supplement: Supplementary file 1 — Supplementary Information [file 41598_2018_25600_MOESM1_ESM.pdf]

Supplementary Information

Contrasting roles for actin in the cellular uptake of cell penetrating peptide conjugates

He, L.[1], Sayers, E.J.[1], Watson, P.[2*], and Jones, A.T.[1*]

[1]Cardiff School of Pharmacy and Pharmaceutical Sciences, Redwood Building, Cardiff University, Cardiff, Wales, CF10 3NB; [2]Cardiff School of Biosciences, Cardiff University, Cardiff, Wales, CF10 3AX.

Supplementary method 1: Expression and purification of recombinant proteins

BL21 (DE3) *E.coli* (Novagen, Feltham, UK) were transformed with a pEV3b plasmids containing His_6-EGFP or His_6-EGFP-R8 transformed cultures were induced using 0.5 mM IPTG (Sigma-Aldrich, Poole, UK) for 3 hr. Proteins were purified under native conditions by affinity chromatography on Ni-NTA (nickel-nitrilotriacetic acid) resin column (Qiagen, Manchester, UK). The purity of the expressed protein was analysed using SDS-PAGE (Supplementary Fig 1A,B) and both proteins were then dialysed overnight against phenol red-free D-MEM in 20 kDa molecular weight cut off Slide-A-Lyzer dialysis cassettes (Life Technologies, Paisley, UK), MALDI-TOF analysis showed the purified EGFP-R8 to have a mass of 29125.00 (Supplementary Fig. 1C). Aliquots (50µl) of 50µM concentration were stored at -80˚C until needed.

Supplementary method 2: Cell permeability assay of A431 cells in presence of inhibitors

A431 cells were seeded on MatTek dishes as described in section 2.4 of main manuscript. On the day of experiment cells were preincubated with the inhibitors as described in section 2.5, with the addition of a DMSO control (0.4% DMSO in serum-free D-MEM (SFM), 45 min pre-incubation), negative control (SFM, 45 min pre-incubation) and a positive control (0.1% Triton X100 in SFM, no preincubation). Cells were then re-incubated with fresh inhibitor, or control solution for 1 hr in the presence of 3 µM DRAQ7 (abcam, Cambridge, UK) to mimic the CPP incubation period. Finally, cells were washed three times in SFM before imaging by confocal microscopy (section 2.6 of main manuscript).

Supplementary Figure 1

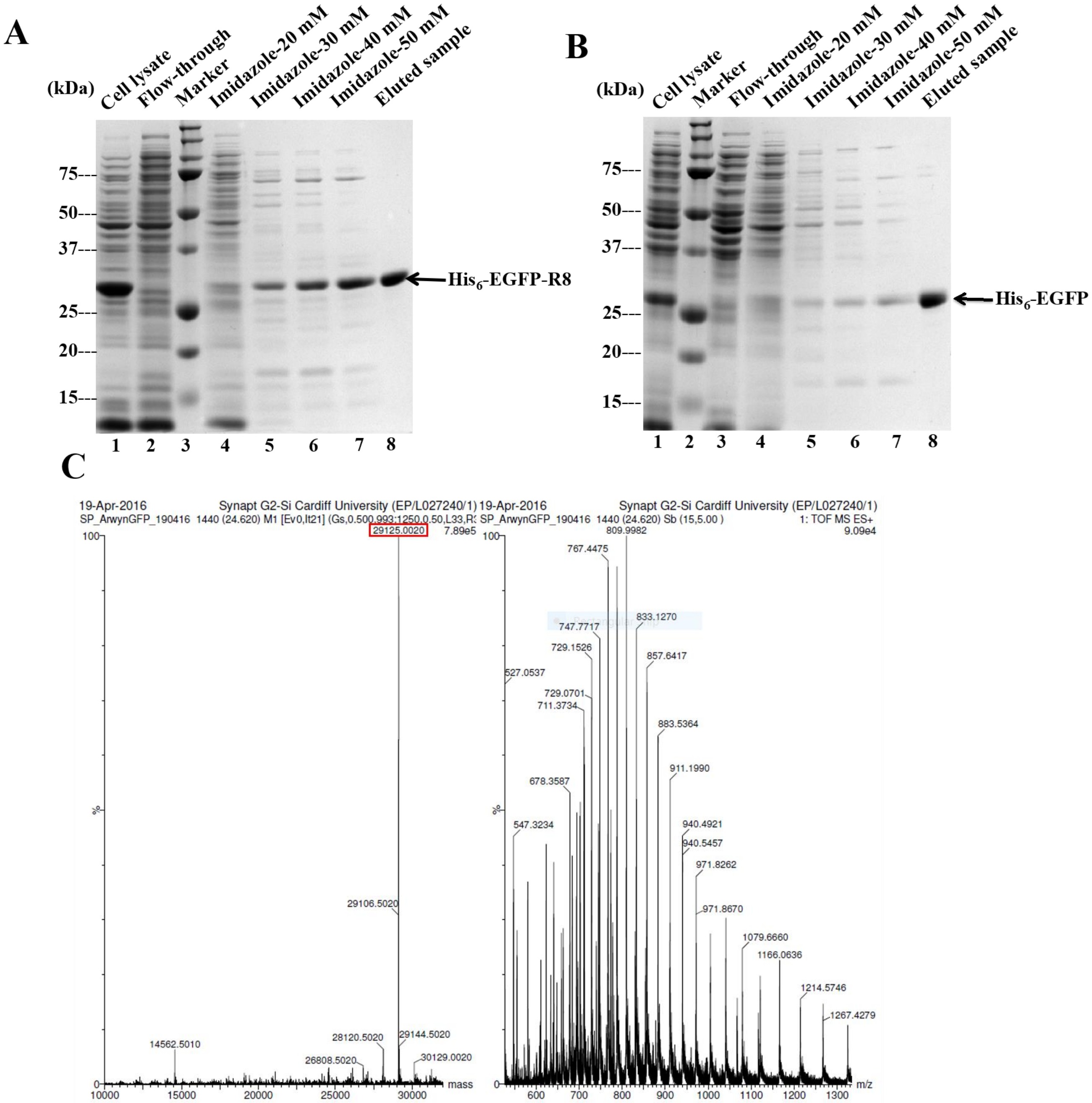

Supplementary Figure 1. Purification of EGFP conjugates. Crude cell lysate (40 µg; lane 1) containing (A) His6-EGFP-R8 or (B) His6-EGFP obtained from a 5 L *E. coli* preparation was applied to an equilibrated Ni-NTA resin column. Samples were collected from the initial Flow-through and then following four washes of buffer (20 mM Tris-HCl pH 7.5, 400 mM NaCl) containing increasing concentrations of 20-50 mM imidazole. The remaining material was eluted with buffer containing 250 mM imidazole and protease inhibitor cocktail. Sample (20 µl) from each wash (lane 4-7) and 2 µg of the eluted His6-EGFP-R8 (A, lane 8) or His6-EGFP (B, lane 8) were run on a 12% SDS-PAGE gel and visualised by Coomassie blue staining. The eluted samples (lane 8) were then dialysed against DMEM. (C) MS Spectra of EGFP-R8 analysed on a Waters Synapt G2-Si mass spectrometer. Left, deconvoluted spectrum (theoretical: 29125.02, observed: 29125.00). Right, ESI-MS before deconvolution.

Supplementary Figure 2

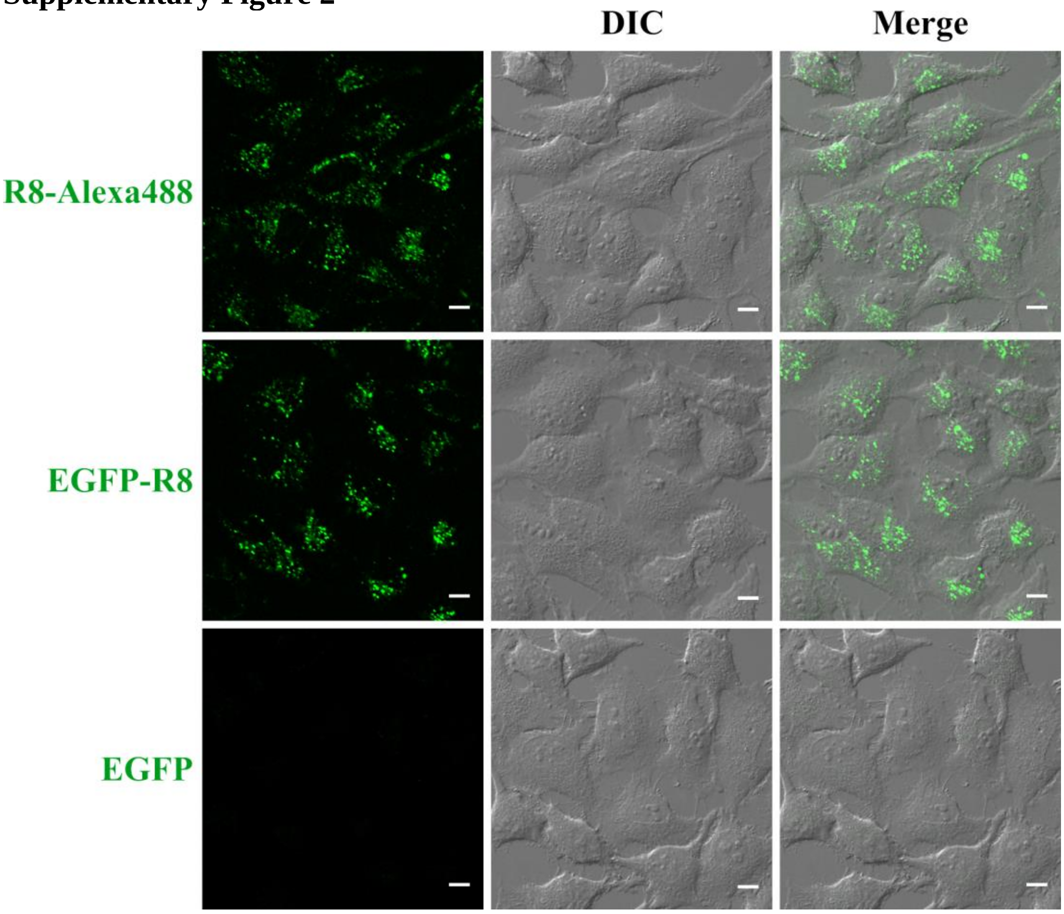

Supplementary Figure 2. Endocytosis of R8-Alexa 488 and EGFP-R8 in HeLa cells. Cells were washed and incubated with 2 µM R8-Alexa 488, EGFP-R8 or EGFP in serum free D-MEM for 1 hr, and analysed by confocal microscopy. Images shown are single sections, DIC profiles and merged overlays. Scale bars 10 µm.

Supplementary Figure 3

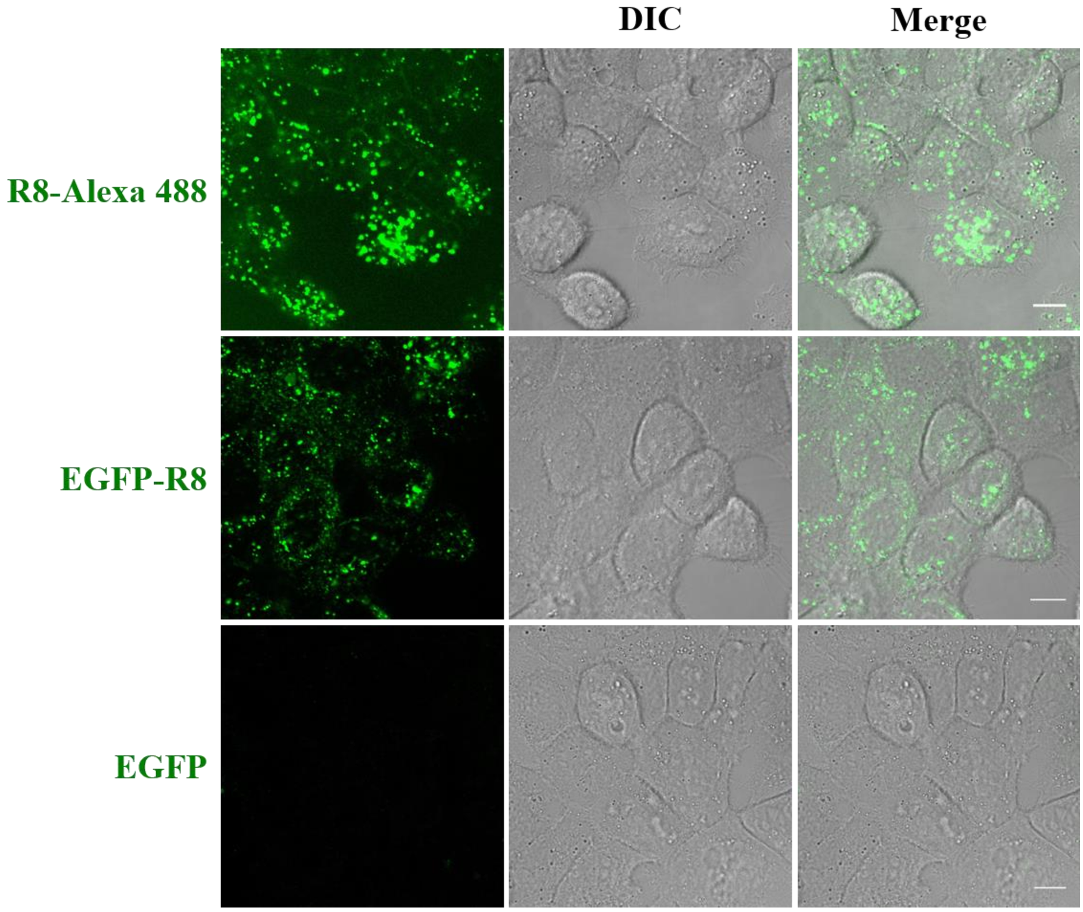

Supplementary Figure 3. Endocytosis of R8-Alexa 488 and EGFP-R8 in A431 cells. Cells were washed and incubated with 2 µM R8-Alexa 488, EGFP-R8 or EGFP in serum free D-MEM for 1 hr, and analysed by confocal microscopy. Images shown are single sections, DIC profiles and merged overlays. Scale bars 10 µm.

Supplementary Figure 4

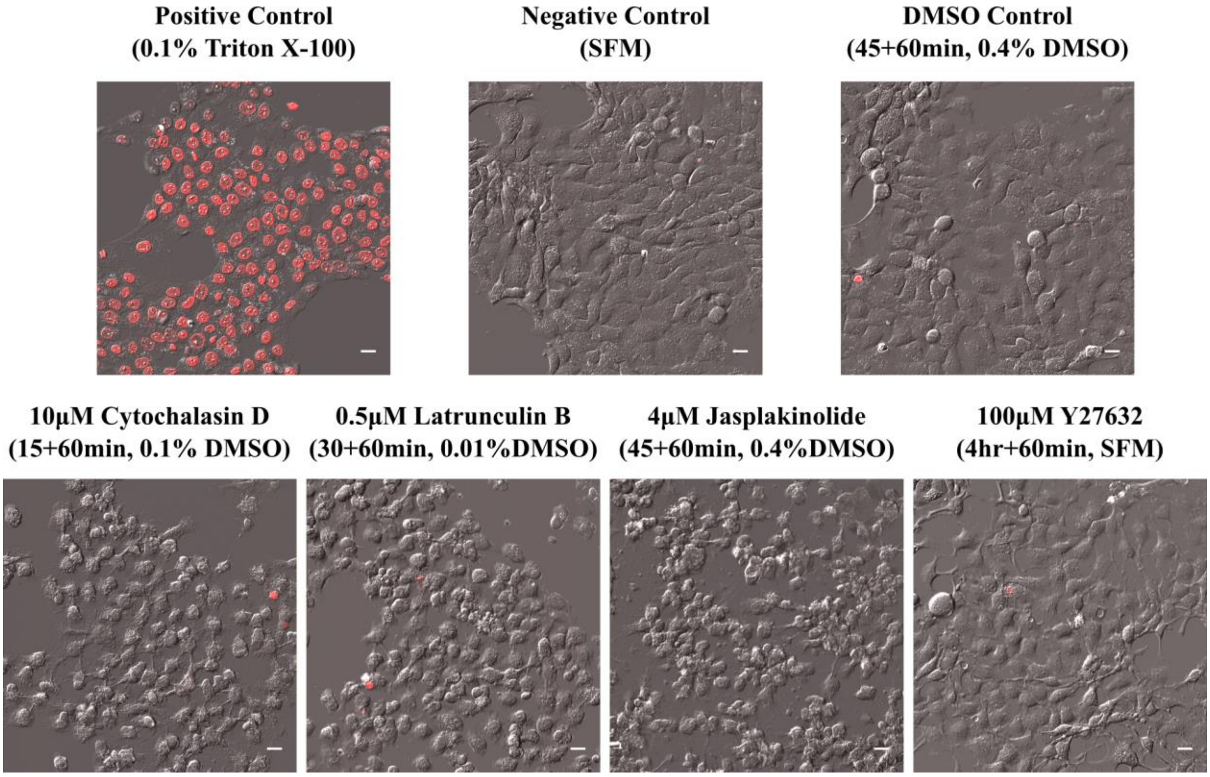

Supplementary Figure 4. Membrane permeability analysis of inhibitors utilised in this study. A431 cells were pre-incubated with the inhibitor for a specified time before being incubated for 1hr in the presence of cell permeation marker DRAQ7 (3 µM, red). Brackets also indicate final concentration of DMSO utilised with inhibitor (Y27632 contained no DMSO). Images represent single sections taken by confocal microscopy using 40X objective, scale bars = 20 µm

Supplementary Figure 5

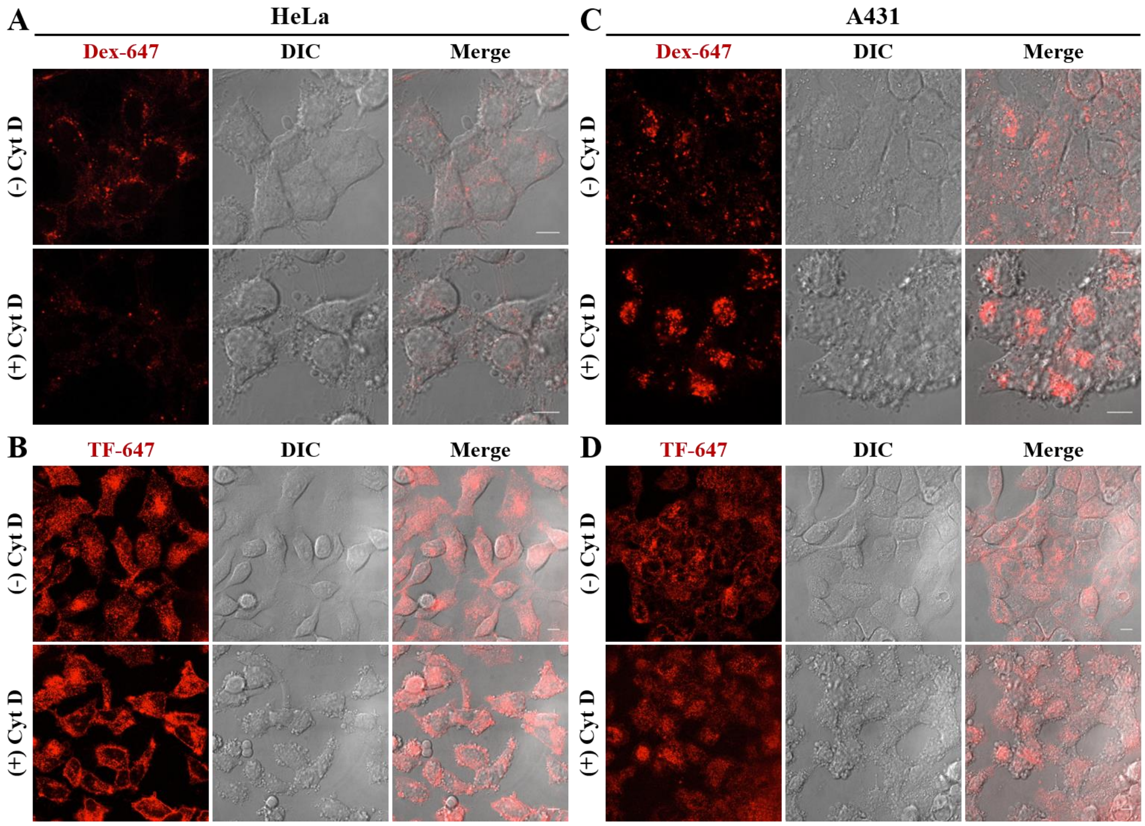

Supplementary Figure 5. Cyt D effects on the cellular uptake of transferrin or dextran in HeLa or A431 cells. HeLa (A, B) and A431 (C, D) cells were pre-treated with 10 µM Cyt D or diluent control for 15 min prior to incubation with 5 µg/ml Transferrin-Alexa647 (B, D TF-647) for 30mins or 0.1 mg/ml 10kD dextran-Alexa 647 (A, C Dex-647) for 1 hr in the absence or presence of 10 µM Cyt D before washing and analysis by confocal microscopy. Shown are single projection images of fluorescence only (TF-647/Dex-647), DIC and merges of fluorescence and DIC of the same cells. Scale bars 10 µm.

Supplementary Figure 6

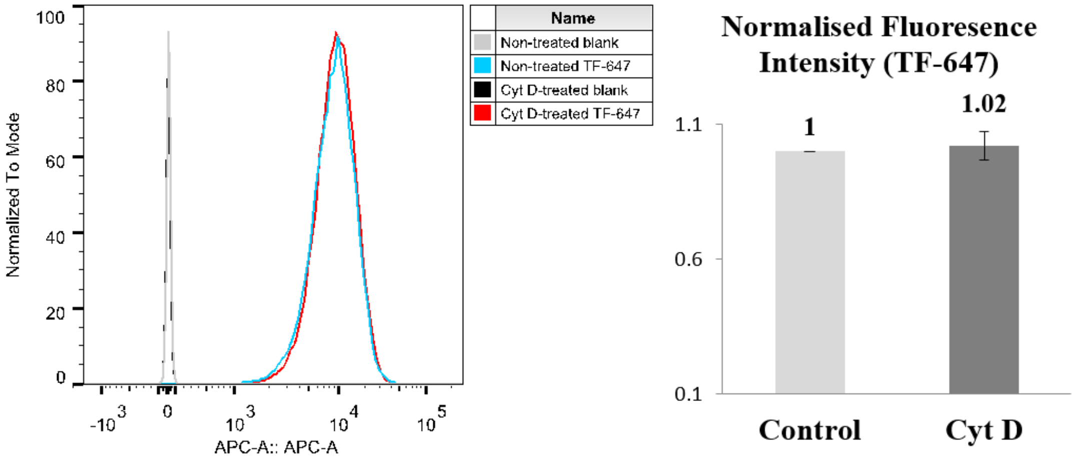

Supplementary Figure 6. Effects of Cyt D treatment on the cellular uptake of Transferrin-Alexa647 (TF-647) by A431 cells. Cells were pre-treated with 10 µM Cyt D or diluent control for 15 min prior to an incubation with 5 µg/ml TF-647 in the absence or presence of 10 µM Cyt D for 30 min. Cells were then washed, collected and analysed by flow cytometry. Data represent the mean of geometric means ± S.D. from three separate experiments.

Supplementary Figure 7

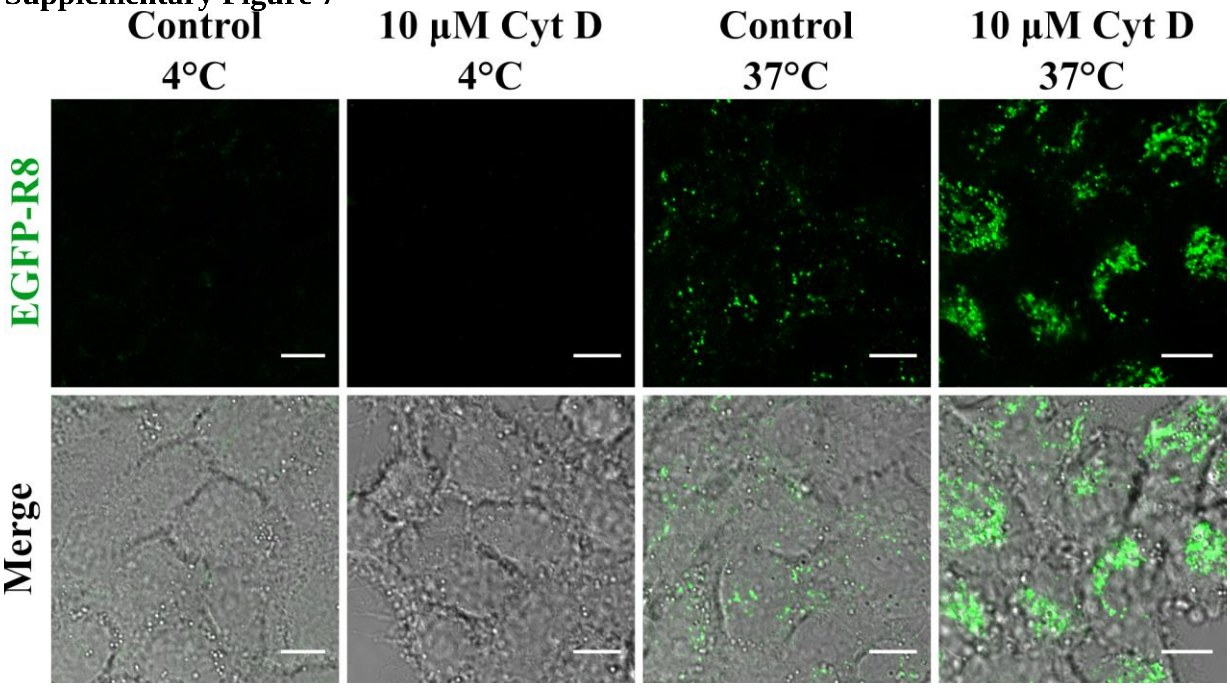

Supplementary Figure 7 Cellular uptake of EGFP-R8 in control or Cyt D-treated A431 cells at 4°C or 37°C. Cells were pre-treated with 10 µM Cyt D or diluent control for 15 min prior to incubation with 2 µM EGFP-R8 in the absence (control) or presence of 10 µM Cyt D for 1 hr at 4°C or 37°C, washed with heparin and analysed using confocal microscopy and shown are single projection images of fluorescence only (top rows) and merges of fluorescence and DIC of the same cells. Scale bars 10 µm.

Supplementary Figure 8

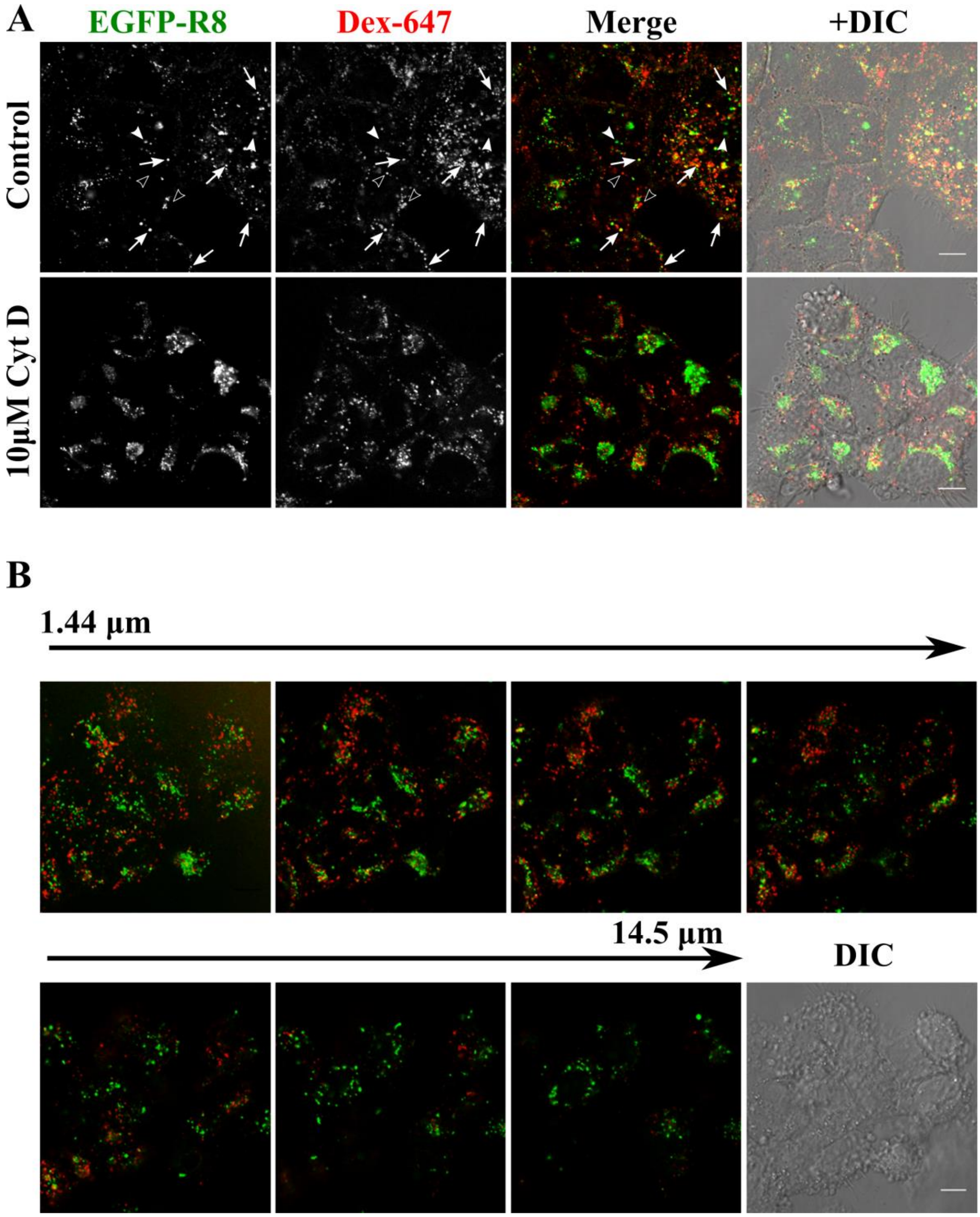

Supplementary Figure 8. Co-localisation of EGFP-R8 and dextran10-Alexa647 (Dex-647) in A431 cells treated with Cyt D. Cells were incubated with 0.1 mg/ml Dex-647 for 2 hr followed by 4 hr chase in fresh growth medium. Cells were then pre-treated with 10 μM Cyt D or diluent control for 15 min prior to incubation with EGFP-R8 (2 μM) for 1 hr in the absence or presence of 10 μM Cyt D. Cell associated fluorescence was analysed using confocal microscopy. (A) Single section images of EGFP-R8 (green) or Dex-647 (red) fluorescence and merges of fluorescence with or without DIC of the same cells. Arrows depict colocalisation, solid arrows depict non-colocalised EGFP-R8 and hollow arrows depict non-colocalised Dex-647. (B) Cells incubated in the presence of 10 μM Cyt D as above, single section images of the Z-axis of the cells from the glass surface to a height of 14.5μm single section images of fluorescence (EGFP-R8, green and Dex-647, red). Scale bars 10 μm.

Supplementary Figure 9

A

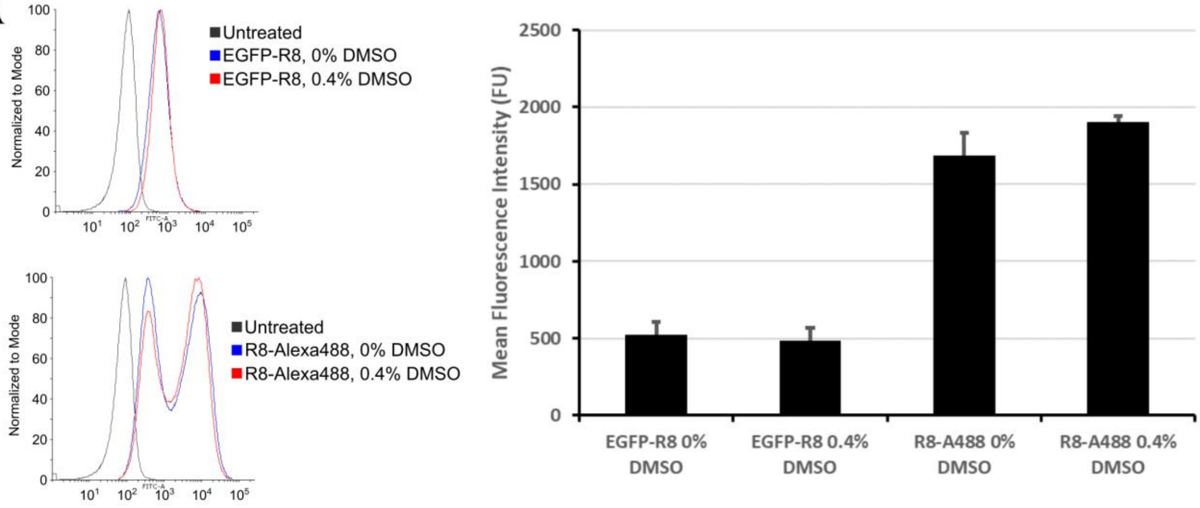

B

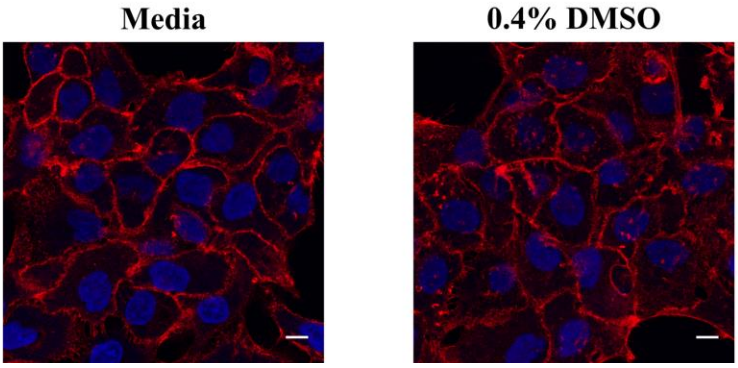

Supplementary Figure 9. Uptake of EGFP-R8 and R8-Alexa488 in the presence and absence of DMSO in A431 cells. (A) Cells were preincubated with 0 or 0.4% DMSO in SFM for 45 mins before re-incubation in 0 or 0.4% DMSO in the presence of either 2 µM EGFP-R8 or 2 µM R8-Alexa488 for 1 hr and analysed by flow cytometry as described in materials and methods of the main manuscript. Data represents the mean of the geomeans ± S.D from three independent experiments. (B) Cells incubated with or without DMSO as above were fixed and stained for actin using rhodamine-phalloidin and nucleus labelled with Hoechst33342. Scale bars = 10 µm

Supplementary Figure 10

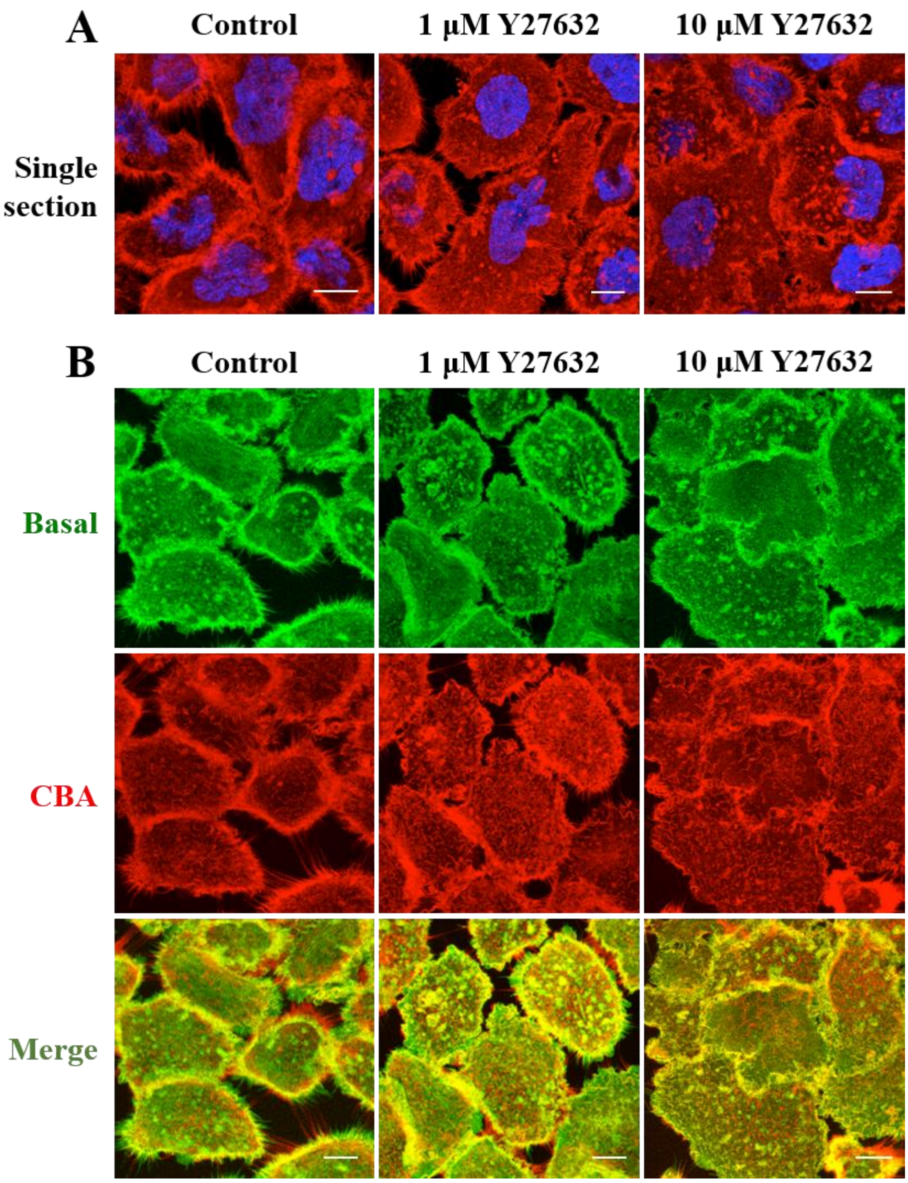

Supplementary Figure 10. Effects of ROCK inhibitor Y27632 (low concentrations) on the actin architecture in A431 cells. Cells on coverslips were treated either with diluent control or 1 or 10 µM Y27632 for 4 hr before fixing and staining with Rh-P and Hoechst. (A) Single sections show the overall distribution of the actin relative to the nucleus. (B) Actin arrangement from the basal, CBA regions and composite (Merge) of basal and CBA. Scale bars 10 µm.
